# Distance Is Brain: Reflections of a Young Neurologist

**DOI:** 10.7759/cureus.112192

**Published:** 2026-07-07

**Authors:** Ereida Rraklli

**Affiliations:** 1 Department of Neurology, Berat Regional Hospital, Berat, ALB

**Keywords:** albania, diagnostic access, neurological diseases, patient-centered approach, timely diagnosis

## Abstract

Neurological care is highly time-sensitive, yet many patients first present to a non-university regional hospital located far from specialized centers. This editorial reflects on the role of regional neurology in the early recognition, assessment, and coordination of care for acute and subacute neurological disorders. Stroke illustrates this challenge most clearly, as delays in recognition, imaging, transfer, and reperfusion treatment may directly affect outcome. However, the same problem extends to other neurological conditions, including suspected multiple sclerosis, spinal cord disease, severe headache, recurrent seizures, and rapidly progressive neuromuscular weakness. In these situations, clinical urgency may be evident before advanced diagnostic resources are available. Non-university regional hospitals, therefore, represent a critical first point in the neurological care pathway rather than a peripheral component of the system. Strengthening referral pathways, emergency neurological training, imaging access, ambulance coordination, communication with tertiary centers, and teleconsultation may reduce the clinical consequences of distance. Improving regional neurological care is not only a logistical need but also a matter of equity, particularly for patients living outside major urban centers.

## Editorial

There are moments in medicine when distance is not measured only in kilometers but in minutes, uncertainty, and responsibility. In a regional hospital in Albania, neurological care often begins far from the centers where the most advanced treatments are available. It begins in a small emergency room, with a frightened family, a patient who may be unable to speak, move, walk, see clearly, or explain what is happening, a computed tomography scan that must be organized quickly, and a decision that cannot wait.

Modern neurology has advanced remarkably. Stroke units, mechanical thrombectomy, magnetic resonance imaging, disease-modifying therapies, neuroimmunology, epilepsy monitoring, neurocritical care, and specialized centers have transformed outcomes for many patients. At the same time, neurological disorders remain a major and growing contributor to global disability and health loss [1]. These advances have changed what neurologists can offer, but they have also made timely access to diagnosis, treatment, and organized care increasingly important. In stroke care specifically, rapid recognition, imaging, treatment eligibility assessment, reperfusion therapy, and coordinated systems of care are central to improving outcomes [2].

In this editorial, “regional neurology” refers to neurological care delivered in a non-tertiary regional or non-university regional hospital, particularly outside specialized urban centers, and includes the early recognition, assessment, stabilization, referral, and coordination of patients within the broader neurological care system.

Yet for many patients, the first neurological decision is still made in a regional hospital. It is made before the patient reaches a tertiary center, before magnetic resonance imaging is available, before all answers are clear, and sometimes before the full clinical picture has declared itself. This first decision may determine whether the patient is treated locally, transferred urgently, observed, or referred through a slower pathway.

In stroke care, we often say that “time is brain.” In regional practice, distance is also brains. In Albania, where stroke-unit care is concentrated mainly in Tirana and at the Memorial Hospital in Fier, regional hospitals may be located approximately 30 to more than 240 km from the nearest stroke center. In my own regional setting, the nearest stroke center is about 60 km away. On a map, this distance may appear modest; at the bedside of a patient with sudden hemiplegia, aphasia, gaze deviation, or reduced consciousness, it feels much longer. It becomes the distance between suspicion and confirmation, transfer and treatment, and hope and disability. Current acute ischemic stroke guidance emphasizes the importance of prehospital evaluation, rapid imaging, acute treatment decisions, and early in-hospital management within organized systems of care [2]. The expansion of mechanical thrombectomy has further increased the need for efficient stroke pathways capable of identifying eligible patients early and connecting them with specialized centers without avoidable delay [3].

However, this reality is not limited to stroke. Stroke is the most visible example because time is so clearly linked to outcome. The same challenge appears throughout neurology. A young patient with visual symptoms, weakness, or sensory complaints may need MRI to confirm or exclude multiple sclerosis. A patient with progressive paraparesis may need urgent spinal imaging to rule out spinal cord compression or inflammatory myelopathy. A patient with a persistent severe headache may require further evaluation even after a normal CT scan. A patient with recurrent seizures may need specialist assessment, electroencephalography, treatment adjustment, or admission to a higher-level center. A patient with rapidly progressive neuromuscular weakness may require urgent recognition before respiratory failure develops. These examples reflect the broader burden of neurological disorders and the need for timely access to neurological diagnosis and care [1].

In these situations, the clinical signs may be present, but the diagnostic pathway may be elsewhere. It may be delayed by distance, referral procedures, ambulance availability, limited imaging access, institutional coordination, or waiting. These delays may also reflect limited availability of CT or MRI technicians outside routine hours, emergency department overcrowding, and uneven access to ambulance resources. The neurological examination may suggest urgency, but the system must also be able to respond with urgency.

The pressure of these moments is difficult to describe. The family looks for certainty. The patient may be unable to speak or may be frightened by sudden weakness, visual loss, severe pain, confusion, or loss of function. The clock is moving. Frontline clinicians in regional hospitals often make urgent decisions with limited information and immediate consequences. The physician must ask quickly: Is this an emergency? Can this patient be managed locally? Is CT sufficient? Is MRI urgently needed? Is this a stroke, a mimic, a spinal lesion, an inflammatory disorder, an infection, a seizure-related event, or another condition? Is the patient stable for transfer? Will the patient arrive in time? These questions are not abstract. They are asked in real time, often with incomplete information, and the answers may shape the rest of a person’s life.

For clinicians working in regional hospitals, this is both a burden and a lesson. It teaches humility, because not every answer is immediately available. It teaches vigilance, because missing a neurological emergency can change a life. It teaches responsibility, because the first decision is often made far from the final destination of care. It also teaches respect for regional medicine, where clinical judgment must often stand before technology arrives.

This reflection is not a criticism of specialized centers or centralized care. No health system can place every advanced neurological service in every hospital. Specialized centers are necessary and lifesaving. They concentrate expertise, imaging, interventional procedures, intensive care, multidisciplinary teams, and rehabilitation pathways. However, the hospitals where patients first arrive are also essential. They provide the first door, the first neurological examination, the first CT scan, the first suspicion, the first explanation to the family, and often the first decision.

Regional neurology should therefore not be viewed as medicine at the margins. It is medicine at the beginning of the pathway. If the first point of care is unsupported, delayed, or isolated, the entire neurological pathway becomes vulnerable. A patient may technically live within reach of advanced treatment but still fail to benefit from it if recognition, referral, imaging, transport, or communication is delayed. Evidence from stroke care has shown that patients treated in non-urban hospitals may experience poorer outcomes and reduced access to key stroke interventions, highlighting the clinical consequences of geographic disparity [4]. In neurology, access is not defined only by the existence of specialized centers. It is defined by whether patients can reach the right care at the right time.

Regional neurology needs practical support. This support must be realistic for regional hospitals and should address not only referral pathways but also staffing, emergency department capacity, imaging availability, and transport coordination. Clear referral pathways are essential. Emergency teams should be trained to recognize neurological emergencies rapidly. Urgent imaging access, including magnetic resonance imaging when clinically indicated, should be organized through defined pathways rather than improvised case by case. Ambulance coordination should be reliable. Communication with tertiary centers should be structured, immediate, and clinically useful. In acute stroke, coordinated systems support rapid assessment, transfer, and treatment decisions [2]. Teleconsultation and telestroke systems may help reduce the clinical consequences of distance by connecting regional hospitals with specialized neurological expertise in real time [5]. These measures do not replace specialized care; they help patients reach it before distance becomes destiny.

The concept of equity in neurology should include patients outside major urban centers. It should include patients not only with acute stroke but also those with suspected multiple sclerosis, spinal cord disease, severe headache, seizures, neuromuscular weakness, and other neurological disorders requiring timely diagnosis and treatment. For these patients, distance may mean delayed diagnosis, delayed treatment, delayed rehabilitation, delayed recovery, and sometimes permanent disability. Given the global burden of neurological disorders, improving access to timely neurological care should be considered an important public health priority [1].

Regional neurology is not secondary neurology. It is the first line of neurological responsibility. In stroke, distance is brains. In broader neurology, distance can also mean lost function, lost independence, lost opportunity, and lost dignity. For patients outside major centers, reducing the weight of distance is not only a logistical challenge but also a clinical need, a public health priority, and a matter of equity.
